# Investigation of Testosterone, Androstenone, and Estradiol Metabolism in HepG2 Cells and Primary Culture Pig Hepatocytes and Their Effects on 17βHSD7 Gene Expression

**DOI:** 10.1371/journal.pone.0052255

**Published:** 2012-12-26

**Authors:** Gang Chen, Sicong Li, Xinxing Dong, Ying Bai, Ailiang Chen, Shuming Yang, Meiying Fang, Galia Zamaratskaia, Olena Doran

**Affiliations:** 1 Key Laboratory of Agro-Product Quality and Safety, Institute of Quality Standards and Testing Technology for Agro-Products, Chinese Academy of Agricultural Sciences (CAAS), Beijing, China; 2 College of Animal Science and Technology, China Agricultural University, Beijing, China; 3 Department of Food Science, Swedish University of Agriculture Science (SLU), Uppsala, Sweden; 4 Centre for Research in Biosciences, Faculty of Health and Life Sciences, University of the West of England, Bristol, United Kingdom; University of Kentucky, United States of America

## Abstract

Steroid metabolism is important in various species. The accumulation of androgen metabolite, androstenone, in pig adipose tissue is negatively associated with pork flavor, odour and makes the meat unfit for human consumption. The 17β-hydroxysteroid dehydrogenase type 7 (17βHSD7) expressed abundantly in porcine liver, and it was previously suggested to be associated with androstenone levels. Understanding the enzymes and metabolic pathways responsible for androstenone as well as other steroids metabolism is important for improving the meat quality. At the same time, metabolism of steroids is known to be species- and tissue-specific. Therefore it is important to investigate between-species variations in the hepatic steroid metabolism and to elucidate the role of 17βHSD7 in this process. Here we used an effective methodological approach, liquid chromatography coupled with mass spectrometry, to investigate species-specific metabolism of androstenone, testosterone and beta-estradiol in HepG2 cell line, and pig cultured hepatocytes. Species- and concentration-depended effect of steroids on 17βHSD7 gene expression was also investigated. It was demonstrated that the investigated steroids can regulate the 17βHSD7 gene expression in HepG2 and primary cultured porcine hepatocytes in a concentration-dependent and species-dependent pattern. Investigation of steroid metabolites demonstrated that androstenone formed a 3′-hydroxy compound 3β-hydroxy-5α-androst-16-ene. Testosterone was metabolized to 4-androstene-3,17-dione. Estrone was found as the metabolite for β-estradiol. Inhibition study with 17βHSD inhibitor apigenin showed that apigenin didn’t affect androstenone metabolism. Apigenin at high concentration (50 µM) tends to inhibit testosterone metabolism but this inhibition effect was negligible. Beta-estradiol metabolism was notably inhibited with apigenin at high concentration. The study also established that the level of testosterone and β-estradiol metabolites was markedly increased after co-incubation with high concentration of apigenin. This study established that 17βHSD7 is not the key enzyme responsible for androstenone and testosterone metabolism in porcine liver cells.

## Introduction

Steroids control the differentiation and proliferation processes of cells and tissues. The steroids biosynthesis and metabolism are important for maintaining the normal physiological function in mammals. Defects in steroid metabolism in human are associated with many different multifactorial diseases like cancer, diabetes, or neurological diseases etc [1]. Testosterone (T) is the main active androgen. It also can be either irreversibly converted to estrogens in the reaction catalyzed by aromatase, or can be transformed to other active form such as dihydrotestosterone [2]. In parallel with active testosterone metabolites, a number of physiologically inactive steroids (pheromones) can also be produced from T. The biosynthesis of active and inactive testosterone metabolites is different between species. For example, boars can produce high amount of pheromone androstenone (5α-androst-16-en-3-one) in their testis, which can then be transported with the blood stream in adipose tissue. Excessive accumulation of androstenone, alongside with skatole in pig adipose tissue results in “boar taint”, an unpleasant odor of some pig meat [3]. Therefore, reduction of androstenone level in pig meat is one of key challenges for pig industry [4].

Accumulation of androstenone in pig tissue can be related either to the high rate of androstenone biosynthesis or low rate of androstenone metabolism or both [5]. Although the mechamisms regulating androstenone biosyntehsis have been extensively studied [6], the pathway for androstenone metabolism and factors influencing the rate of androstenone metabolism remain unclear. Furthermore, one of the main research questions is how to reduce androstenone levels in meat without affecting other androgen-regulated anabolic functions [7].

The enzyme family of hydroxysteroid dehydrogenases (HSDs) plays the key role in the reduction and oxidation of steroid hormones, which play essential role in the transformation of inactive steroids to their active forms [8], or are responsible for phase I steroid metabolism [2]. The HSDs distribution and enzymatic activity varies in different tissues. It has been reported that HSDs are highly expressed in steroidogenic tissues and mainly catalyze the biosynthesis of active steroids [8], also they were abundantly expressed in peripheral tissue such as in liver [8]. Previous study using pig primary hepatocytes reported the involvement of 3βHSD enzyme in androstenone metabolism [5], [9]. However, the role of other key HSDs enzymes, such as 17βHSD, in the hepatic androstenone metabolism remains unclear. Our previous study on pigs found a negative association between the hepatic 17βHSD7 gene expression and the level of fat androstenone [10]. Therefore, it is of interest to investigate further the role of 17βHSD7 in steroids metabolism in pigs. It is known that the steroids metabolic pathway differs between species [11]. Although the family of HSD enzymes is known to be involved in metabolism of steroids in a number of species, including human and pig [8], [10], the species-specific involvement of specific HSD isoforms in the hepatic metabolism of sex steroids is not clear and need to be studied.

Investigating the role of HSD enzymes in steroid metabolism is complicated by limitation of the analytical methods and approaches which are generally used for this type of studies. Currently, the analysis of steroids is mainly conducted by immunoassays, which are rapid and highly sensitive, but non-specific methods [12]. It has also been reported that the steroid values obtained by immunoassays are generally overestimated [13]. The gas chromatography mass spectrometry (GC-MS) has frequently been used as “golden standard” for steroids analysis. However, the main disadvantage of GC-MS is that this is a time consuming approach, and the unavoidable sample derivatization limits its further application, especially in the “metabolomics” for detection of the changes in metabolites profile and for identification of the “unknown metabolites” without reference standards [14]. The evolution of detection capability of liquid chromatography coupled with tandem mass spectrometry (LC-MS/MS) has been greatly improved during the past years [15]. Nowadays, the LC-MS/MS holds a great potential for improving and moving forward steroids metabolic study.

The aim of this study was to investigate species-specific steroid metabolism using human HepG2 cell line and isolated primary pig hepatocytes via employing an advance LC-MS/MS techniques. The effect of steroids to 17βHSD7 gene expression regulation was also studied.

## Materials and Methods

### Chemicals and Reagents

Testosterone (T), androstenone (A), and β-estradiol (E2) were from Sigma-Aldrich (Shanghai, China). Stock solutions of the steroids were prepared in methanol at concentrations of 1 g/L. Working solutions of steroids were prepared by diluting the stock solutions in methanol. All other reagents and solvents (HPLC grade) were purchased from Fisher Scientific (service in Beijing, China). Deionized water was obtained using Millipore water purification system (Millipore, France).

All the reagents for molecular biology study were purchased from TAKARA and Promega. Collagenase was purchased from Sigma (Shanghai, China). Cell culture medium and other cell culture reagents were purchased from Hyclone (Beijing, China). Oligonucleotides and related DNA fragments were synthesized in Sangon Company (Shanghai, China).

### Experimental Material

In order to compare the differences of steroids metabolism and 17βHSD7 gene expression between species, human liver carcinoma cell line (HepG2), and isolated primary porcine hepatocytes were used for this study. Previous studies reported abundant expression of 17βHSD7 gene in the above cells [10], [16]. The HepG2 cell line was purchased from Peking Union Medical College (PUMC, Beijing, China). Primary porcine hepatocytes were isolated from Large White male pigs (age 3–5 days), which were bought from Shunxinlong pig farm (Beijing, China).

### Isolation and Culturing of Pig Hepatocytes

All the experimental designs involving animals were approved by the Animal Ethics Committee of the China Agricultural University, Beijing, China (permission number: 2011-11-23-1). The pigs have been fasting for 24 h before surgery. After anesthetization with 1.5–1.7 ml/kg BW of 3% of pelltobarbitalum natricum, the liver was taken out from abdominal cavity. The portal cannula was placed then the liver was perfused for 15 min with D-hanks buffer at 37°C at a flow rate of 30 mL/min. The liver was further purfused for 2 min with D-hanks buffer containing 0.5% collagenase, and then taken out after dissecting the surrounding tissues. After 10 min digestion, the liver capsule was disrupted and its parenchyma was suspended in Hanks buffer. The cell suspension was filtered and washed twice by centrifugation at 500 rpm for three min. Cell viability was assessed by 0.2% trypan blue exclusion and was greater than 90% in all cases. Approximately 5×10^6^ cells were plated into 10 cm Petri dishes with 10 ml of Dulbecco modifed Eagle’s medium (DMEM) containing 20% fetal bovine serum (FBS) supplemented with insulin (10 mg/L), penicillin (100 U/mL) and streptomycin (100 µg/mL). The hepatocytes were incubated overnight in a humidified chamber maintained at 37°C with 5% of CO_2_.

### Culturing of HepG2 Cells

This study used the cell culturing procedure described by the Global Bioresource Center (ATCC) with the following modifications: HepG2 cell line was cultured in DME medium supplemented with 15% FBS in 10-cm Petri dishes (Nunc, Denmark) in humidified atmosphere with 5% CO_2_ at 37°C. When the cells reach approximately 90% of confluency, they were re-suspended using trypsine, and were plated in 6-well plates for 24 h prior to steroids treatment.

### Steroids Treatment

The same procedure was used for treatment of primary pig hepatocytes and HepG2 cells. The cells were washed for three times with phosphate buffer solution (PBS) before the treatment. The cell culture medium contained phenol red-free DMEM with 10% of charcoal treated FBS. For the study of species- and concentration-dependent effect of steroids on 17βHSD7 gene expression, The steroids A and T were added to the medium at the final concentrations 0 (control), 1, 10, 100, and 500 nM, respectively. The final concentration of methanol in the incubation was less than 0.1%. In addition, in some cases the cells were treated with a combination of A (1 and 10 nM) and E2 (10 nM) or T (1 and 10 nM) and E2 (10 nM), in order to investigate the 17βHSD7 gene expression under the interaction of androgen and estrogen. Estrogen level applied was to simulate the normal physiological concentration. Hepatocytes were harvested after incubation for 24 h in the presence of individual steroid or their combination. Total RNA was extracted from the cultured cells for expression study.

In order to further investigate and/or confirm the involvement of 17βHSD7 in steroids metabolism, the HepG2 cell line was chosen for inhibition study. HepG2 cells were incubated with 500 nM of A, T, or E2 for 24 h. The 17βHSDs enzymes inhibitor apigenin was added at a concentration 50 µM to control, or in combination with the steroids. Cytotoxicity study showed that apigenin at 50 µM didn’t have any cytotoxic effect to HepG2 cells. The cell culture medium was collected for steroids metabolites analysis. The experiments were repeated for three different days. Triplicates were done in each experiment.

### Isolation of Total RNA and Reverse Transcription

Total RNA was extracted from primary pig hepatocytes and HepG2 cells by using the TRNzol reagent (TIANGEN, Beijing) according to the manufacturer’s instructions. The concentration of total RNA was quantified at 260 nm using the NanoDrop spectrophotometer (ND-2000, Thermo). The quality of total RNA was analyzed by 1% agarose gel electrophoresis. Only the samples which showed integral RNA bands were selected for reverse transcription. Reverse transcription was performed using the ImProm-II™ Reverse Transcriptase Kit (Promega, Beijing) and 1 µg of total RNA in 20 µL of RNase free water was applied accordingly.

### Real-Time PCR

Real-Time PCR was carried out using cDNA as a templates and the SYBRGreen PCR Master Mix (TIAGEN, Beijing) according to the manufacturer’s protocol. The primers for 17βHSD7 target gene and for housekeeping internal control gene (hypoxanthine phosphoribosyltransferase, HPRT) for human and pig samples are listed in Table 1. All primers were designed using Primer 5.0 software (PREMIER Biosoft International, Palo Alto, CA, US). The related sequences were downloaded from NCBI gene bank.

**Table 1:** Real-time PCR primer sequences for amplification of 17βHSD7 gene, and for housekeeping gene hypoxanthine phosphoribosyltransferase (HPRT).

Gene	Species	Forward primer, 5′-3′	Reverse primer, 5′-3′	Product bp
17βHSD7	Human	GCACATTAGGGTCACTATTCA	AGGGCTCACTATGTTTCTCA	146
17βHSD7	Pig	ACATCCAGCACAGCAAAGG	GCATCCACATAAAAGGAGATAAAAT	178
HPRT	Human pig	CAGTCAACGGGCGATATAAAAGT	CCAGTGTCAATTATATCTTCAACAATCA	95

Real-time PCR was performed using Bio-Rad Real-Time CFXTM96 System (Applied Biosystems, Foster, CA, US). Each reaction well contained 9 µL of 2.5×RealMasterMix/20×SYBR solutions, 0.05 µM of primers (forward and reverse), 1 µL of cDNA, and 9.8 µL of H_2_O. The final reaction volume was 20 µL. All samples were run in triplicate. The following PCR conditions were used: the initial denaturation at 95.0°C for 4 min, and then 39 cycles of denaturation at 95.0°C for 30 s, annealing at 60.5°C for 30 s, and extension at 72°C for 30 s. The last stage for the melt curve was as follows: 95.0°C for 10 s, from 65.0°C to 95.0°C, increment 0.5°C for 5 s. The relative quantification of 17βHSD7 gene expression was calculated using the standard curve-based method for relative RT-PCR [17].

### Mass Spectrometry Condition

Cultured medium from the cells incubated with or without steroids, was collected for analysis of steroid metabolites. The analysis was done using liquid chromatography coupled with mass spectrometry. Two types of mass spectrometry were applied: 1) QSTAR® Elite with a TurboIonSpray® electrospray ion source, and controlled by Analyst QS 2.0 software (AB Sciex, ON, Canada); 2) API 5000 with a Turbo VTM electrospray ion source, and controlled by Analyst 1.4.2 software (AB Sciex, ON, Canada). The HPLCs for both mass systems were Agilent 1200 SL, equipped with a binary pump, vacuum solvent degasser, column oven and autosampler (Agilent Technologies, Waldbronn, Germany).

The chromatographic separation for both LC-MS/MS systems was performed on the Zorbax Eclipse Plus C18 column (2.1×100 mm, 3.5 µm) with a guard column (2.1×12.5 mm, 5 µm) (Agilent Technologies, USA). The flow rate was set to 300 µL/min. For the +ESI mode, the mobile phase was 0.1% formic acid in water (A) and methanol (B), and for the -ESI mode, the mobile phase was water (A) and methanol (B). In order to improve the negative ionization efficiency, 30% of ammonium solvent was infused into the mass spectrometry at the -ESI mode with a flow rate 5 µL/min using a Harvard syringe pump (Holliston, MA, USA) with a T connection post column. A linear gradient program was used for both LC-MS/MS systems: 0–5 min from 50 to 95% B; 5–9 min, 95% B; 9–9.1 min, 95 to 50% B; 9.1–15 min, 50% B. Sample injection volume was 20 µL for QSTAR® Elite, and was 5 µL for API 5000.

The QSTAR® Elite system was applied for identifying the steroids metabolites. The ESI source was run on both positive and negative mode. Spectrometry parameters were listed in supplementary material (Table S1). The Q1 mass monitoring used scan mode at the range from 200 to 600 m/z. Mass intensity above 100 counts was set for acquiring product ions on TOF analyzer. An information dependent acquisition (IDA) method was set to optimize data acquisition. All the gas was supplied with nitrogen (purity ≥99.995%).

The API 5000 system with ESI source was applied for further steroids metabolites characterization. The spectrometry parameters were listed in supplementary material (Table S2). Analytes were monitored by multiple reactions monitoring (MRM), and the precursor and product ions information was obtained from the QSTAR® Elite system. The same nitrogen gas as above was supplied. Mass selection for Q1 and Q3 analyser was set on unit resolution.

### Sample Extraction

Sample extraction was performed using 8 mL of the cultured medium. To achieve the required volume, the cultured media from same treatment was pooled together. The extraction procedure was as follows: samples were mixed with tri-chloric acid (TCA) to the final concentration of 1% in order to precipitate protein, and then centrifuged at 17226 g (12000 rpm) for 10 min. The supernatants were collected and purified with solid phase extraction (SPE) on Bond Elut-C18 extraction cartridges (500 mg/3 cc, Agilent, Lake Forest, CA, USA). The solvent volume for SPE cartridge preconditioning, washing steps, and sample elution steps were optimized to reduce the solvent waste and give optimal clean-up efficiency. Cartridges were conditioned with 3 mL methanol and equilibrated with 3 mL water, followed by sample loading. Cartridges were then washed with 3 mL water and 3 mL 5% of methanol in water, respectively. After drying the cartridges for 5 min under vacuum, the T and E2 were eluted by addition of two times of 3 mL ACN, and A was eluted by addition of three times of 3 mL ACN. The elution solvents were evaporated under nitrogen at 40°C, and reconstituted with 400 µL of 62.5% methanol (MeOH). Solvents were passed through 0.22 µm filter paper (Jinteng company, Tianjin, China), and then transferred to inserts for mass analysis.

For the SPE extraction procedure validation, fractions of the sample loading solvents and washing solvents were collected, dried under nitrogen, and reconstituted. No target steroids were found in the analyzed fractions. The recoveries of the spiked steroids in blank medium and extracted using the above procedures were: A 59.1%, T 93.3%, E2 81.3%. The samples were analyzed in triplicates.

### Statistical Analysis

Data was analyzed by the Statistical Analysis System, version 9.0 (SAS Institute, Cary, NC, USA). Anova procedure was used to evaluate the group difference, and the means of groups were compared by Duncan’s multiple comparison. A value of P<0.05 was regarded as statistically significant. The values of the relative quantity of gene expression were presented as the mean ± SD.

## Results

### Effect of A and A Plus E2 on 17βHSD7 Gene Expression

**Figure 1:**
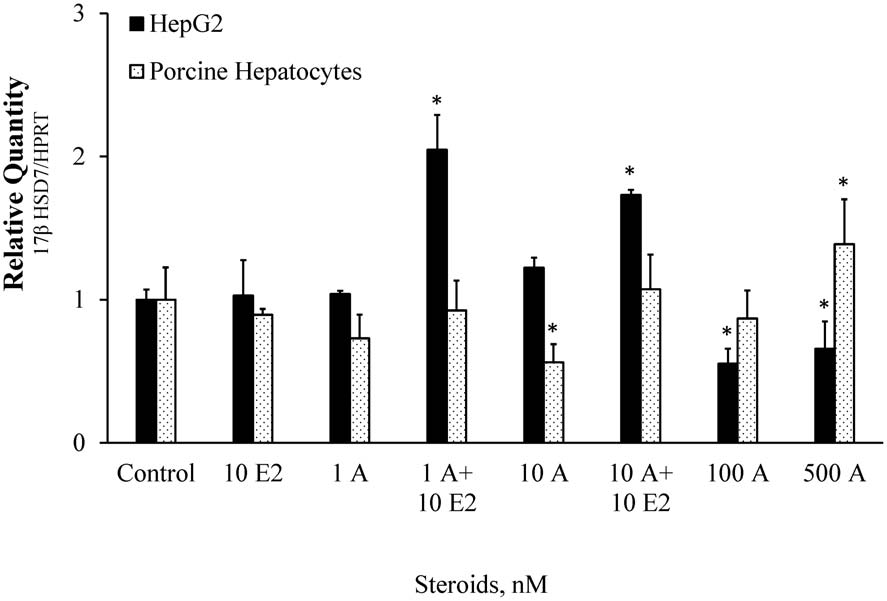
Effect of androstenone or androstenone in combination with β-estradiol on 17βHSD7 gene expression in HepG2 cell line and pig primary hepatocytes. The data were normalized with HPRT housekeeping gene. Treatments: 1A = 1 nM androstenone, 1A +10E2 = 1 nM androstenone +10 nM β-estradiol, 10A = 10 nM androstenone, 10A +10E2 = 10 nM androstenone +10 nM β-estradiol, 100A = 100 nM androstenone, 500A = 500 nM androstenone. Each bar represents an average value for four independent experiments. Each measurement was done in triplicate. Error bars represent standard errors for means. The effect of treatments to 17βHSD7 gene expression were compared with control, and bars with superscript differ significantly (P<0.05). “Control” cells were kept under same conditions as the experimental cell lines but without added steroids. Incubation time was 24 hr. * indicates p<0.05.

Real-time PCR analysis showed that in cultured HepG2 cells, 1 nM and 10 nM of A did not affect the 17βHSD7 gene expression when compared to control. However, when the cells were treated with A in combination with 10 nM E2, there was a significant induction of 17βHSD7 gene expression in both cases, 1 nM and 10 nM of androsteone (by 104 and 73% respectively when compared to control). Increasing the A concentration to 100 nM and 500 nM resulted in inhibition of 17βHSD7 gene expression by 45 and 34% respectively (Figure 1).

The 17βHSD7 gene expression in pig primary hepatocytes exhibited different pattern compared with HepG2 cell line. There was no effect of 1 nM A (either in absence or presence of E2) on the gene expression. Increasing A concentration to 10 nM resulted in significant decrease (by 44%) in 17βHSD7 expression in porcine hepatocytes, but only in case when E2 was not added. No significant effect of A was observed at concentration of 100 nM, and there was 39% activation of 17βHSD7 gene expression in the presence of 500 nM A (Figure 1).

### Effect of T and T Plus E2 to 17βHSD7 Gene Expression

**Figure 2:**
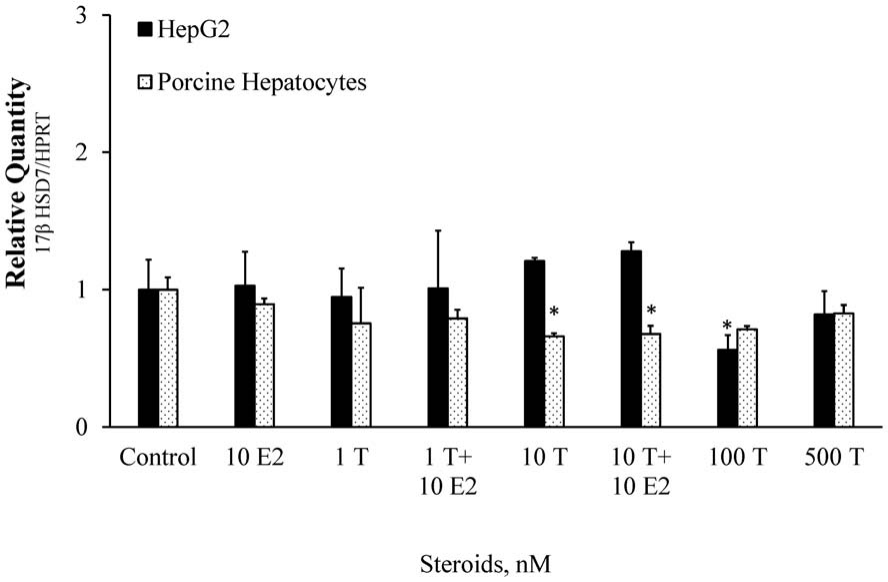
Effect of testosterone or testosterone in combination with β-estradiol on 17βHSD7 gene expression in HepG2 cell line and pig primary hepatocytes. The data were normalized with HPRT housekeeping gene. Treatments: 1T = 1 nM testosterone, 1T +10E2 = 1 nM testosterone +10 nM β-estradiol, 10T = 10 nM testosterone, 10T +10E2 = 10 nM testosterone +10 nM β-estradiol, 100T = 100 nM testosterone, 500A = 500 nM testosterone. Each bar represents an average value for four independent experiments. Each measurement was done in triplicate. Error bars represent standard errors for means. The effect of treatments to 17βHSD7 gene expression were compared with control, and bars with superscript differ significantly (P<0.05). “Control” cells were kept under same conditions as the experimental cell lines but without added steroids. Incubation time was 24 hr. * indicates p<0.05.

Treatment of HepG2 cells with T did not affect significantly 17βHSD7 gene expression when compared with the control. The only exception was the treatment with T at concentration of 100 nM, where 17βHSD7 expression was significantly reduced by 44% (Figure 2). In contrast to A, co-incubation of HepG2 cells in the presence of T and 10 nM of E2 didn’t affect the 17βHSD7 gene expression.

In the case of pig primary hepatocytes, the 17βHSD7 gene expression was not affected significantly by 1 nM, 100 nM and 500 nM of T. Reduction in 17βHSD7 gene expression (by 34%) was observed in presence of 10 nM T. Co-incubation the primary hepatocytes with 1 nM T and 10 nM E2 did not have any effect on 17βHSD7 gene expression, whilst co-incubation with 10 nM T and 10 nM E2 significantly reduced 17βHSD7 gene expression by 32% (Figure 2).

### Steroids Metabolism Study

In this part of our study we aimed (i) to compare the metabolites of selected steroids (A, T, and E2) in HepG2 and cultured pig hepatocytes. This was achieved by using an advanced time of flight mass spectrometry; and (ii) to determine species-specific involvement of 17βHSDs enzymes in hepatic steroid metabolism. This was achieved via enzyme inhibition study specific to 17βHSDs enzymes.

### Studies on A Metabolism

**Figure 3:**
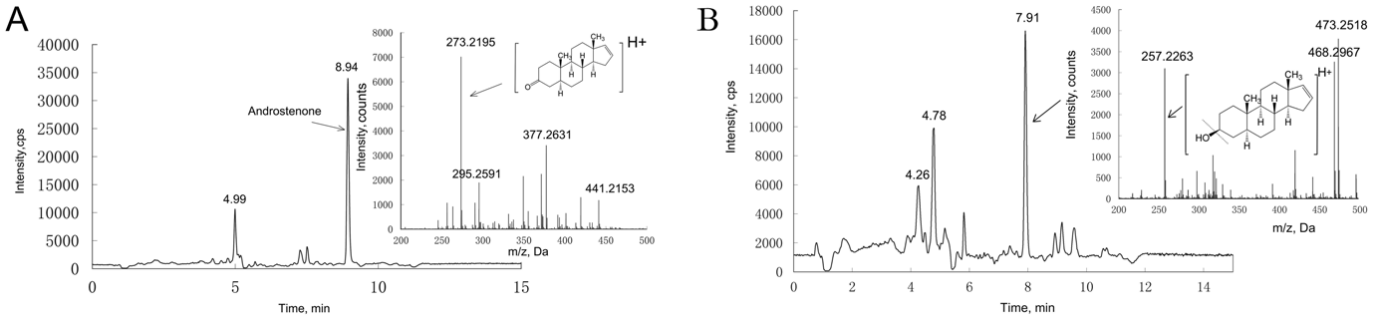
Chromatogram and mass spectra of androstenone and metabolite. The (A) represents androstenone in the medium before cell culture, and the (B) represents identified androstenone metabolite in the medium after cell culture.

After incubation of 500 nM of A with HepG2 or primary pig hepatocytes, the abundance of A precursor ion (C19H28O; MH+ = 273.2195) was significantly reduced (Figure 3A, Figure S1) compared with before incubation. One metabolite ion (m/z = 257.2) was formed in both human and pig hepatocytes. Its retention time (RT) was 1.03 min earlier than that for A (7.91 vs. 8.94 min) (Figure 3B). No metabolite ion was observed in control samples (medium before incubation or incubated medium without androstenone) (Figure S2 and S3). The fragment ions pattern was obtained from TOF (Figure S4) and further confirmed by API5000 at MRM mode. Molecular structure speculation showed that the metabolite ion (m/z 257.2) was from A which lost one oxygen molecule. However, the advent of earlier retention time of the metabolite suggests its polarity increased after metabolism. Therefore, we suggest that the 3-keto group of A was metabolized to 3-hydroxy group in cultured medium. In this case the metabolite structure would match the 3-androstenol structure (m/z 275.2). It was established that the metabolite loses one water molecule in the ion source under the high temperature (500°C) and under high voltage condition. Further identification of the metabolite by infusing 3α-hydroxy-5α-androst-16-ene reference standard didn’t match the retention time of A metabolite. Therefore we speculate the A metabolite was 3β-hydroxy-5α-androst-16-ene. We did not identify any other A metabolites under these experimental conditions.

### Studies on T Metabolism

**Figure 4:**
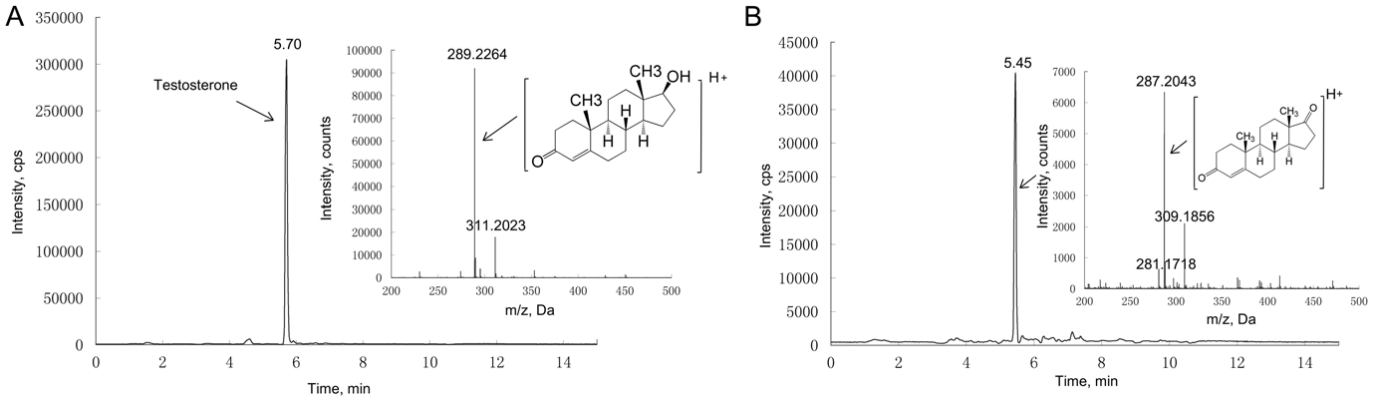
Chromatogram and mass spectra of testosterone and metabolite. The (A) represents testosterone in the medium before cell culture, and the (B) represents identified testosterone metabolite in the medium after cell culture.

Nearly 95% of T (C19H28O2; MH+ = 289.2261) was metabolized in HepG2 cell line when incubated with T concentration of 500 nM (Figure 4A, Figure S5). Identification process of T metabolites found a de-hydronated compound (m/z = 287.2043, RT = 5.45 min, Figure 4B). No metabolite ion was found in control incubation (Figure S6 and S7). By using the purchased reference standard, we further confirmed that the chromatogram and mass spectra of the T metabolite matches the pattern of 4-androstene-3,17-dione (C19H26O2; MH+ = 287.2072, Figure S8, S9 and S10), which is T de-hydronated at C-17 position. The metabolism of T in pig primary hepatocytes demonstrated the similar pattern compared with HepG2 cell line, and the same metabolite was formed in the presence of 500 nM T. Moreover, the primary pig hepatocytes exhibited higher T metabolism potency than HepG2 cell line, and nearly 99% of T was metabolized.

### Studies on E2 Metabolism

**Figure 5:**
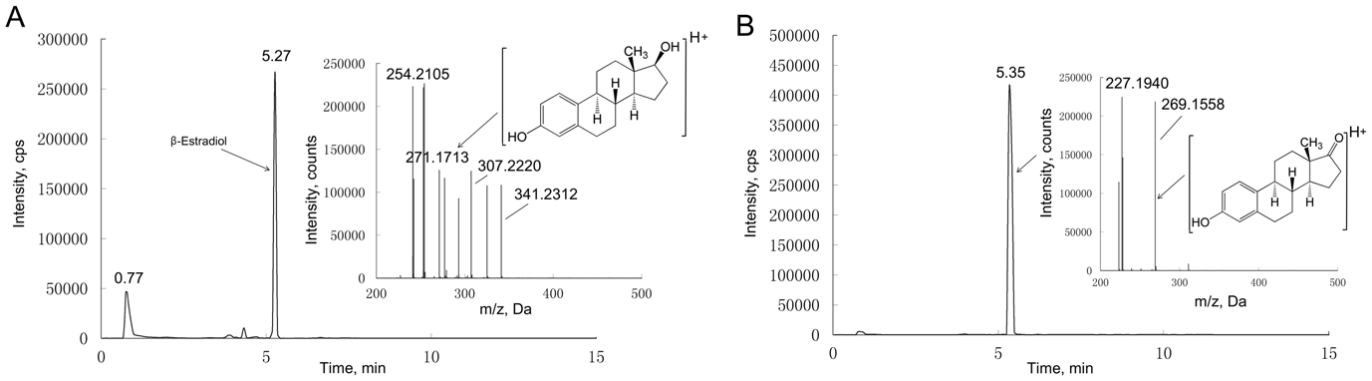
Chromatogram and mass spectra of β-estradiol and metabolite. The (A) represents β-estradiol in the medium before cell culture, and the (B) represents identified β-estradiol metabolite in the medium after cell culture.

Nearly 99% of E2 (C18H24O2; MH+ = 271.1713) was metabolized by the HepG2 cell and primary pig hepatocytes when 500 nM E2 concentration was used (Figure 5A, Figure S11). One E2 metabolite with m/z = 269.1558 was found in both HepG2 cells and primary cultured pig hepatocytes (Figure 5B). No E2 metabolite was found in the control incubation (Figure S12 and S13). The chromatogram and product ions pattern of the metabolite matches that of estrone (C18H22O2; MH+ = 269.1567), when comparing with reference standard (Figure S14, S15 and S16). The metabolite was confirmed as E2 de-hydronated at C-17 position.

### 17βHSD7 Inhibition Study using Apigenin

To determine whether 17βHSD7 is involved in steroids metabolism in hepatocytes, we performed the inhibition study using apigenin, which mainly inhibits 17βHSD1 activity, but also has inhibitory effects on other 17βHSD isoforms. Apigenin at concentration 50 µM were used for 17βHSD7 inhibition study. Results showed that in the cultured HepG2 cell line, A metabolism was not altered by apigenin (Table 2). Apigenin at 50 µM showed very weak inhibition to T metabolism, but this inhibition value was below the equipment detection limit and therefore was viewed as negligible. Apigenin at 50 µM notably inhibited E2 metabolism in the HepG2 cells, the remained E2 was two times more in the medium with apigenin than that without apigenin (Table 2). Surprisingly, we observed increase in the amount of T and E2 metabolites when the cells were incubated in the presence of steroids and apigenin. The amount of metabolites of T and E2 was almost doubles in the presence of high concentration of apigenin (50 µM, Table 2).

**Table 2:** Peak heights of the chromatograms of added steroids precursor ions and their metabolites in the cell culture medium.

Treatment Compounds	NC	C	C-50 µM api
Androstenone	9.5	0.17	0.15
Androstenone metabolite	N.D.	0.2	0.3
Testosterone	12	N.D.	0.18
Testosterone metabolite	N.D.	2.2	4.2
β-estradiol	49	1.8	5.5
β-estradiol metabolite	N.D.	46	79

The values are from the cell culture medium of three replicates equally pooled together.

NC = peak heights of the chromatograms of added steroids and their metabolites before cell culture; C = peak heights of the chromatograms of added steroids and their metabolites after cell culture; C-50 µM api = peak heights of the chromatograms of added steroids and their metabolites under the incubation with 50 µM of apigenin after cell culture. The peak height values are presented after division by 10000 (cps/10000).

N.D. = the values are undetectable.

## Discussion

It is known that 17βHSD7 enzymes play key role in steroids biosynthesis and metabolism [8]. Liver is one of the main organs responsible for steroids metabolism. Previous studies reported results on the expression of 17βHSD7 in the liver of human and pig [8], [10]. However, the mechanism underlying these variations in the expression and the function of the hepatic 17βHSD7 has not been fully understood. Most of the studies related to 17βHSD7 were carried on in steroidogenic tissues. In this study, we observed that the effect of sex steroids A, T or their combination with E2 on hepatic 17βHSD7 gene expression varies depending on the species whether the cells derived from human or pig. Interestingly, co-incubation of E2 with A induces the 17βHSD7 gene expression in cells of human origin, but not in cells of pig origin, whereas co-incubation of E2 with T does not have any stimulatory effect. The effect of A and T on 17βHSD7 gene expression was not only species-depended, but also concentration-dependent. High levels of A induced the 17βHSD7 gene expression in pig primary hepatocytes, whereas inhibited gene expression in HepG2 cells. The observed in this study concentration-dependent and species-dependent variations suggested that pig hepatocytes have higher tolerance to A than HepG2 human cancer cell line. On the other hand, it is important to point out that the cancer cell line may have different physiological function when compared to primary hepatocytes. T at high concentration inhibited 17βHSD7 gene expression in pig primary hepatocytes, this is consistent with our previous study that high level of T was correlated with low levels of 17βHSD7 gene expression in pig liver in vivo [10]. The interaction between steroids and 17βHSD7 gene expression is hard to be explained. It has been reported that 17βHSD7 expression exhibited tissue and developmental-specific manner [8], and there are several steroids related transcription factors have been identified to specifically regulate 17βHSD7 expression in steroidogenic tissue during developmental period. However, there is very limited information regarding regulation of 17βHSD7 gene expression in pig liver. In our recent study using luciferase activity assay and EMSA, we found a steroid-related TF (TALE) in the porcine liver anchoring −850 to −868 bp of promoter region of 17βHSD7 gene [18]. A is generally known as a pheromone and does not have any hormonal function in mammals [19]. However, one of the studies reported that A and other androstane, but not true androgens, can inhibit orphan nuclear receptor CAR-β, one of the main TFs regulating steroidogenase gene expression [20]. The mechanism for specific regulation of 17βHSD7 gene by steroids in liver in different animals needs to be further investigated.

In this study, we found that all the steroids investigated can be metabolised in the cultured liver cell (both HepG2 and isolated primary pig hepatocytes). It is known that the 17βHSD family regulates availability of both androgens and estrogens by catalyzing interconversion of active and inactive forms of steroids [21]. 17βHSD7 were reported to convert estrone to more active estradiol in ovary, uterus placenta [22], and human breast cancer cells [23]. In our study we found inconsistent result that estradiol was converted to estrone in human and porcine liver cells. This is not consistent with data of the literature which showed that 17βHSD3 catalyzes metabolism of androstenedione to T in testicular cells, and that the process is irreversible [24]. The discrepancy between our results and data of the literature suggests the catalytic function of the hepatic 17βHSD7 is different from that in other organs. This suggestion is in a line of the review by Payne et al. (2004) which reported that 17βHSD enzymes catalysis exhibits tissue- and puberty developmental- specificity [8].

In our study, we also demonstrated that 17βHSD7 is not responsible for any of the A and T metabolism under *in vitro* condition, based on the results of the enzyme inhibition study using apigenin. Apigenin partially inhibited the formation of E1 converted from E2, but only at high concentration. Taking into account that apigenin IC_100_ for inhibiting 17βHSD1 enzyme is 50 µM, the concentration which we used might not have been sufficient for inhibiting 17βHSD7 enzyme activity. Although apigenin mainly inhibit 17βHSD1 enzyme function, it is also known as an inhibitor to all of other 17βHSD enzymes including 17βHSD7, although to a less extent [25]. Our inhibition study did not inhibit A and T metabolism in cultured hepatocytes, therefore we suggested that the enzymes of 17βHSD family are not involved in A and T metabolism. Surprisingly, we found a substantial increase in the level of T and E2 metabolites after co-incubation with high concentration of apigenin. This phenomenon is difficult to explain. It has been reported that hepatic steroid metabolism consists of phase I and phase II reactions [26]. We speculate that apigenin might inhibit the phase II metabolism of steroids and causes accumulation of phase I metabolites.

Androstenone was metabolized to more hydrophilic compound, possibly the 3β-androstenol, in both, HepG2 and primary cultured pig hepatocytes. Enzymes responsible for porcine A metabolism have been pursued for decades, since the A accumulation in pork is directly linked to an unpleasant odor and flavor of pork. Reduction/removal of A from pork through activation of the hepatic A metabolism is one of the aims of genetic selection [7]. Previous studies reported a range of enzymes which might be important for the porcine hepatic androstenone metabolism including hydroxysteroid sulfotransferase [27], 3βHSD1 [4], [5], [9], [10], 17βHSD7 [10]. In our study, we only found the presence of the hydroxyl metabolite of A in primary pig heaptocytes, and the conversion of keto- to hydro- group suggests the involvement of HSD enzymes. Since our data indicate that 17βHSD7 enzyme are unlikely to be involved in A metabolism, it points us in the direction that 3βHSD is likely to be the key enzyme which catalyzes the porcine hepatic A metabolism.

### Conclusions

The investigated steroids can regulate the 17βHSD7 gene expression in HepG2 and primary cultured porcine hepatocytes in a concentration-dependent and species-dependent pattern. The steroids can be metabolized in both cell culture models used in this study. In contrast to the previous finding that porcine 17βHSD7 gene expression and steroids levels are closely associated, this study provides evidence that 17βHSD7 is not the key enzyme responsible for androstenone and testosterone metabolism in porcine liver cells.

## Supporting Information

Figure S1 **Chromatogram of androstenone in the medium after cell culture.** It can be seen the androstenone level reduced more than 90% (comparing from peak height) after 24 hr cell culture.
(TIF)

Figure S2 **Chromatogram of androstenone metabolite in the medium before cell culture.** It can be seen that no metabolite was found before incubation.
(TIF)

Figure S3 **Chromatogram of androstenone metabolite in the medium without androstenone after cell culture.** It can be seen that no metabolite was found in the blank medium.
(TIF)

Figure S4 **Product spectra of identified androstenone metabolite in the medium after cell culture.**
(TIF)

Figure S5 **Chromatogram of testosterone in the medium after cell culture. It** can be seen the testosterone level reduced more than 95% (comparing from peak height) after 24 hr cell culture.
(TIF)

Figure S6 **Chromatogram of testosterone metabolite in the medium before cell culture.** It can be seen that no metabolite was found before incubation.
(TIF)

Figure S7 **Chromatogram of testosterone metabolite in the medium without testosterone after cell culture.** It can be seen that no metabolite was found in the blank medium.
(TIF)

Figure S8 **Product spectra of identified testosterone metabolite in the medium after cell culture.**
(TIF)

Figure S9 **Chromatogram and mass spectra of androstendione standard in pure solvent.** It can be seen that the chromatogram and mass spectra of testosterone metabolite match that of the androstendione standard.
(TIF)

Figure S10 **Product spectra of androstendione standard in pure solvent.** The product ion pattern of testosterone metabolite matches that of the androstendione standard.
(TIF)

Figure S11 **Chromatogram of β-estradiol in the medium after cell culture.** It can be seen the β-estradiol level reduced more than 99% (comparing from peak height) after 24 hr cell culture.
(TIF)

Figure S12 **Chromatogram of β-estradiol metabolite in the medium before cell culture.** It can be seen that no metabolite was found before incubation.
(TIF)

Figure S13 **Chromatogram of β-estradiol metabolite in the medium without β-estradiol after cell culture.** It can be seen that no metabolite was found in the blank medium.
(TIF)

Figure S14 **Product spectra of identified β-estradiol metabolite in the medium after cell culture.**
(TIF)

Figure S15 **Chromatogram and mass spectra of estrone standard in pure solvent.** It can be seen that the chromatogram and mass spectra of β-estradiol metabolite match that of the estrone standard.
(TIF)

Figure S16 **Product spectra of estrone standard in pure solvent.** The product ion pattern of β-estradiol metabolite matches that of the estrone standard.
(TIF)

Table S1 **QSTAR® Elite mass spectrometer parameters**
(DOC)

Table S2 **API 5000 mass spectrometer parameters**
(DOC)
