# Supplementary figures and images for: Investigation of Testosterone, Androstenone, and Estradiol Metabolism in HepG2 Cells and Primary Culture Pig Hepatocytes and Their Effects on 17βHSD7 Gene Expression
Source: PLoS One. 2012 Dec 26;7(12):e52255. doi: 10.1371/journal.pone.0052255 (PMC3530596; doi:10.1371/journal.pone.0052255)

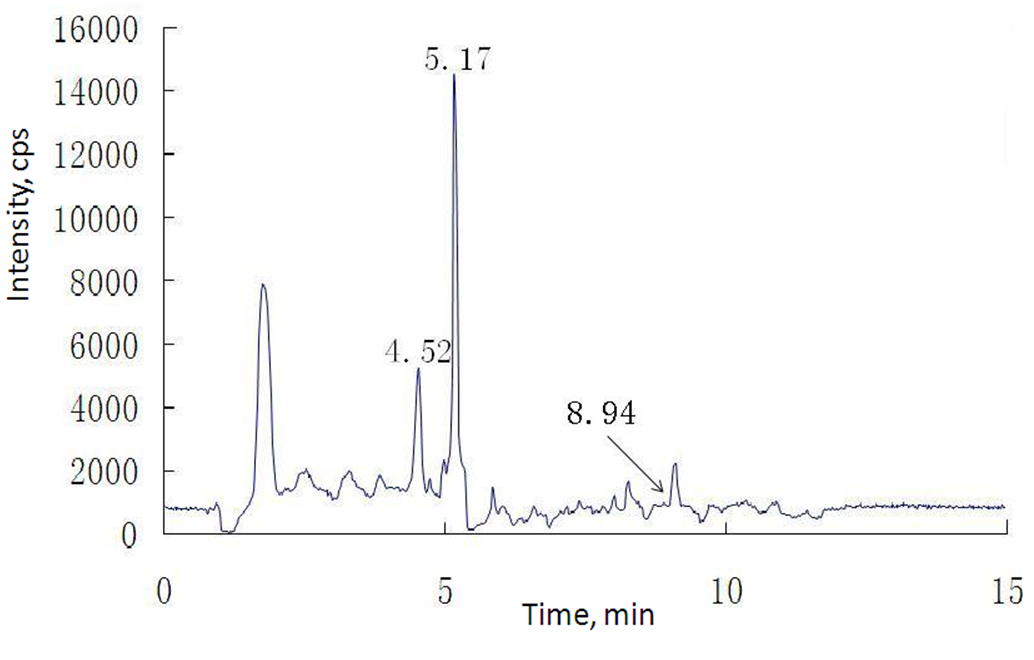

Supplement: Figure S1 — Chromatogram of androstenone in the medium after cell culture. It can be seen the androstenone level reduced more than 90% (comparing from peak height) after 24 hr cell culture. (TIF) [file pone.0052255.s001.tif]

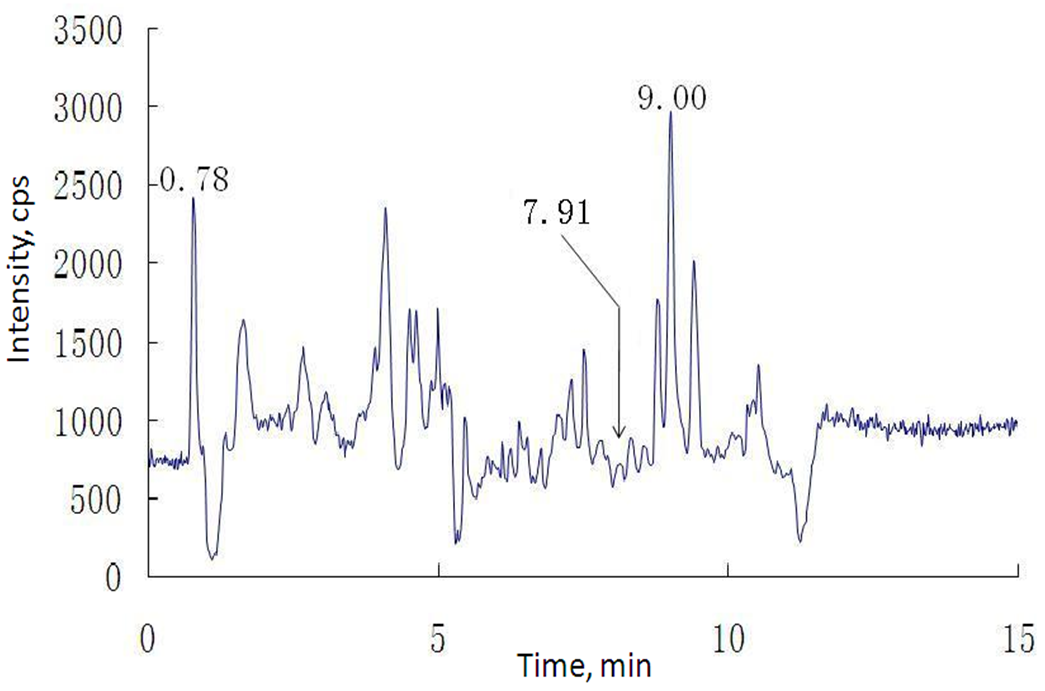

Supplement: Figure S2 — Chromatogram of androstenone metabolite in the medium before cell culture. It can be seen that no metabolite was found before incubation. (TIF) [file pone.0052255.s002.tif]

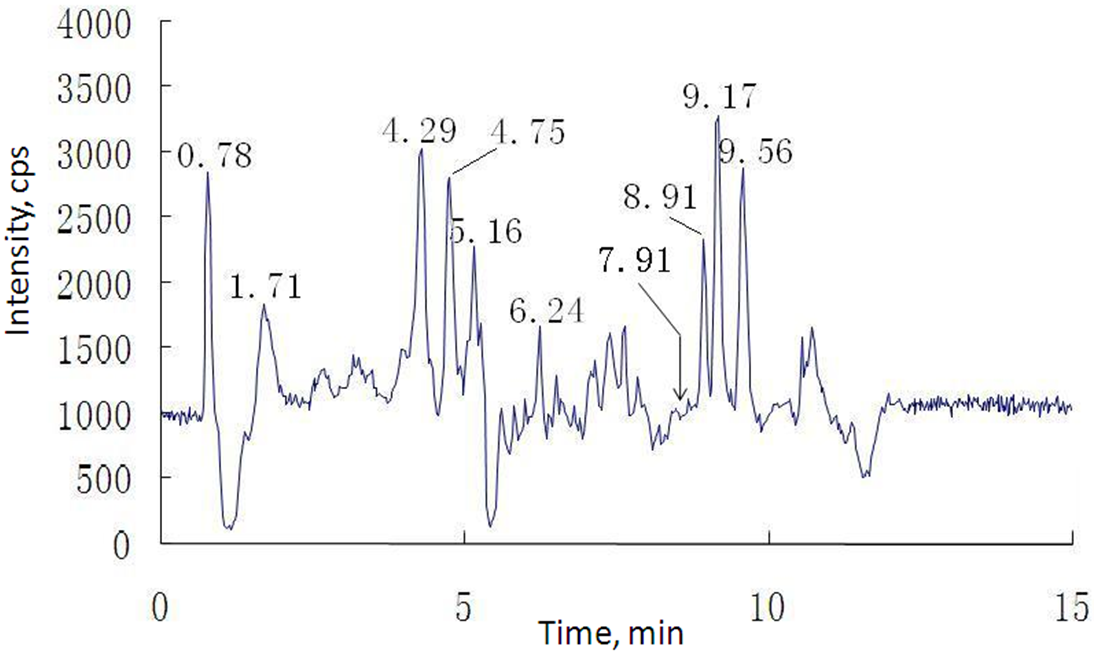

Supplement: Figure S3 — Chromatogram of androstenone metabolite in the medium without androstenone after cell culture. It can be seen that no metabolite was found in the blank medium. (TIF) [file pone.0052255.s003.tif]

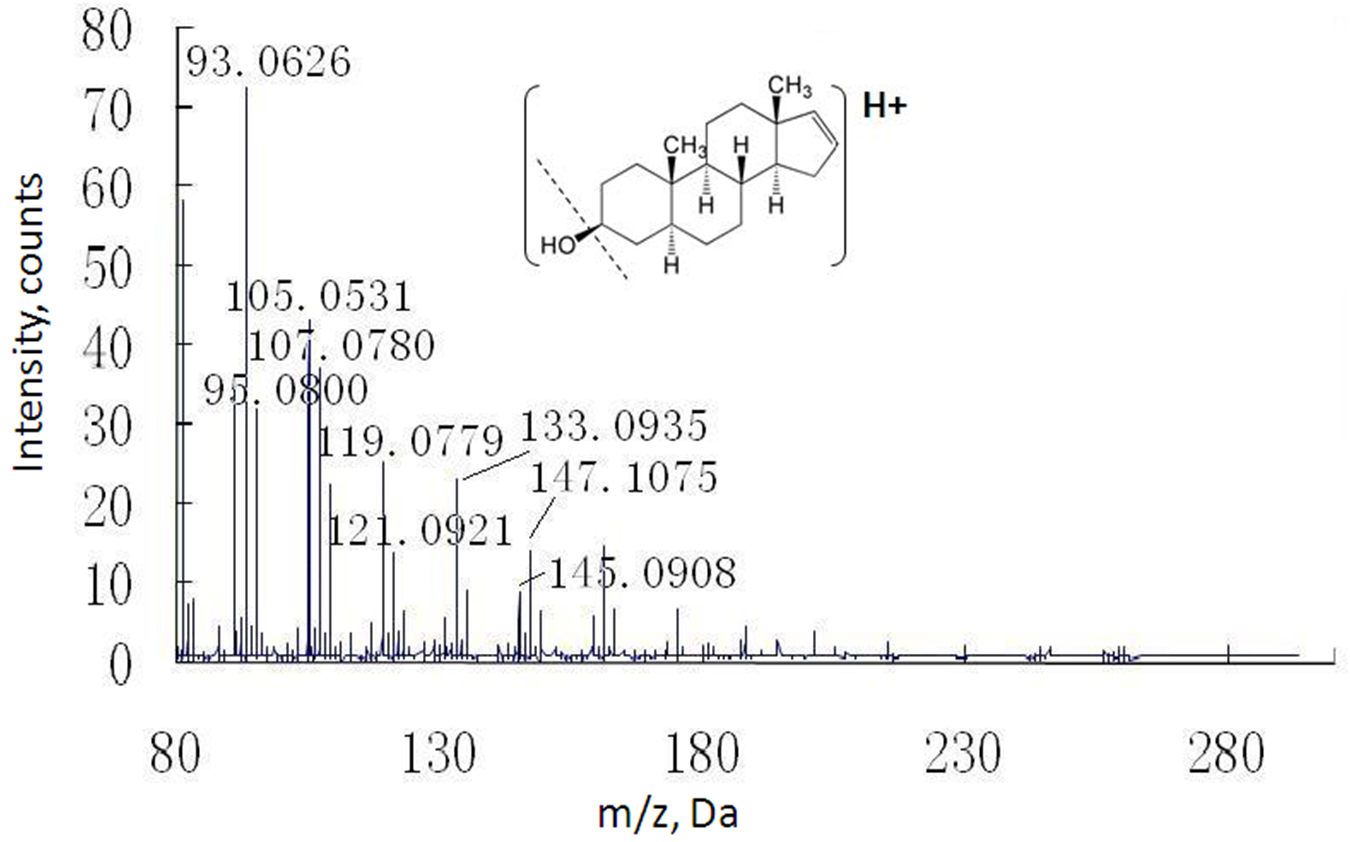

Supplement: Figure S4 — Product spectra of identified androstenone metabolite in the medium after cell culture. (TIF) [file pone.0052255.s004.tif]

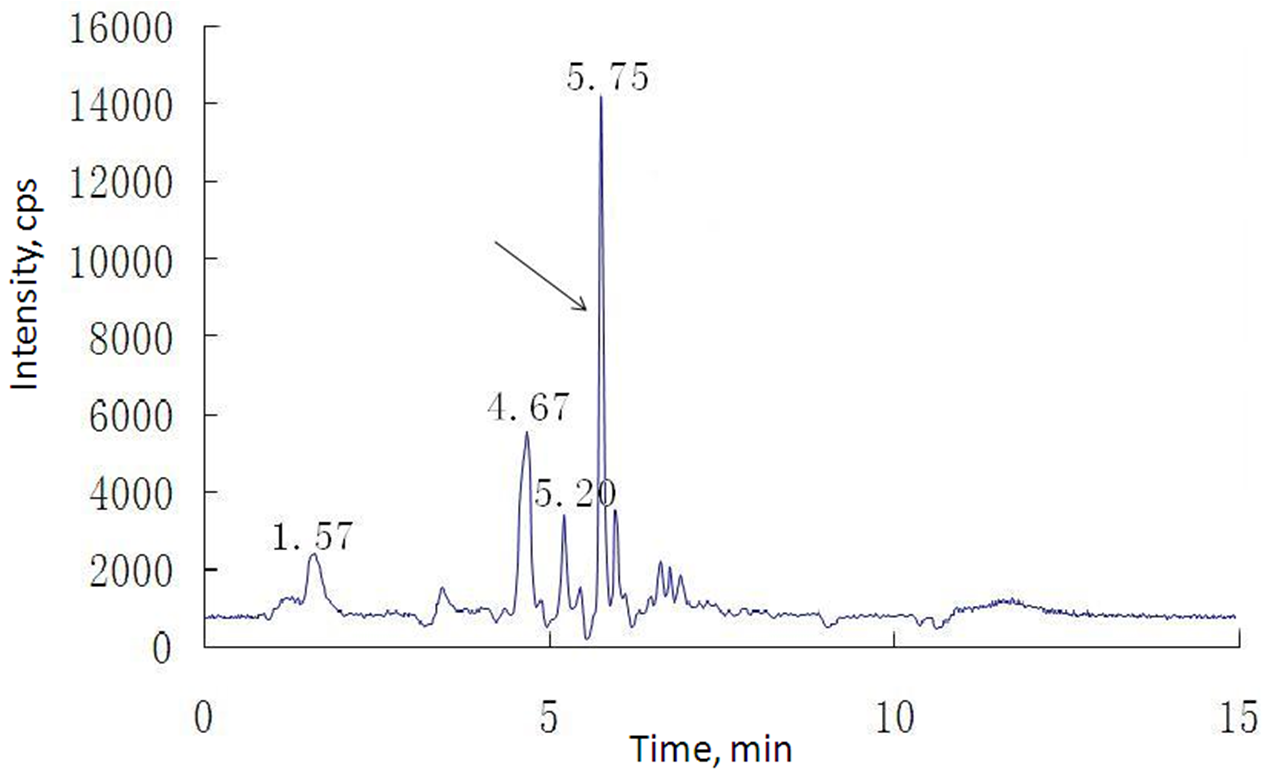

Supplement: Figure S5 — Chromatogram of testosterone in the medium after cell culture. It can be seen the testosterone level reduced more than 95% (comparing from peak height) after 24 hr cell culture. (TIF) [file pone.0052255.s005.tif]

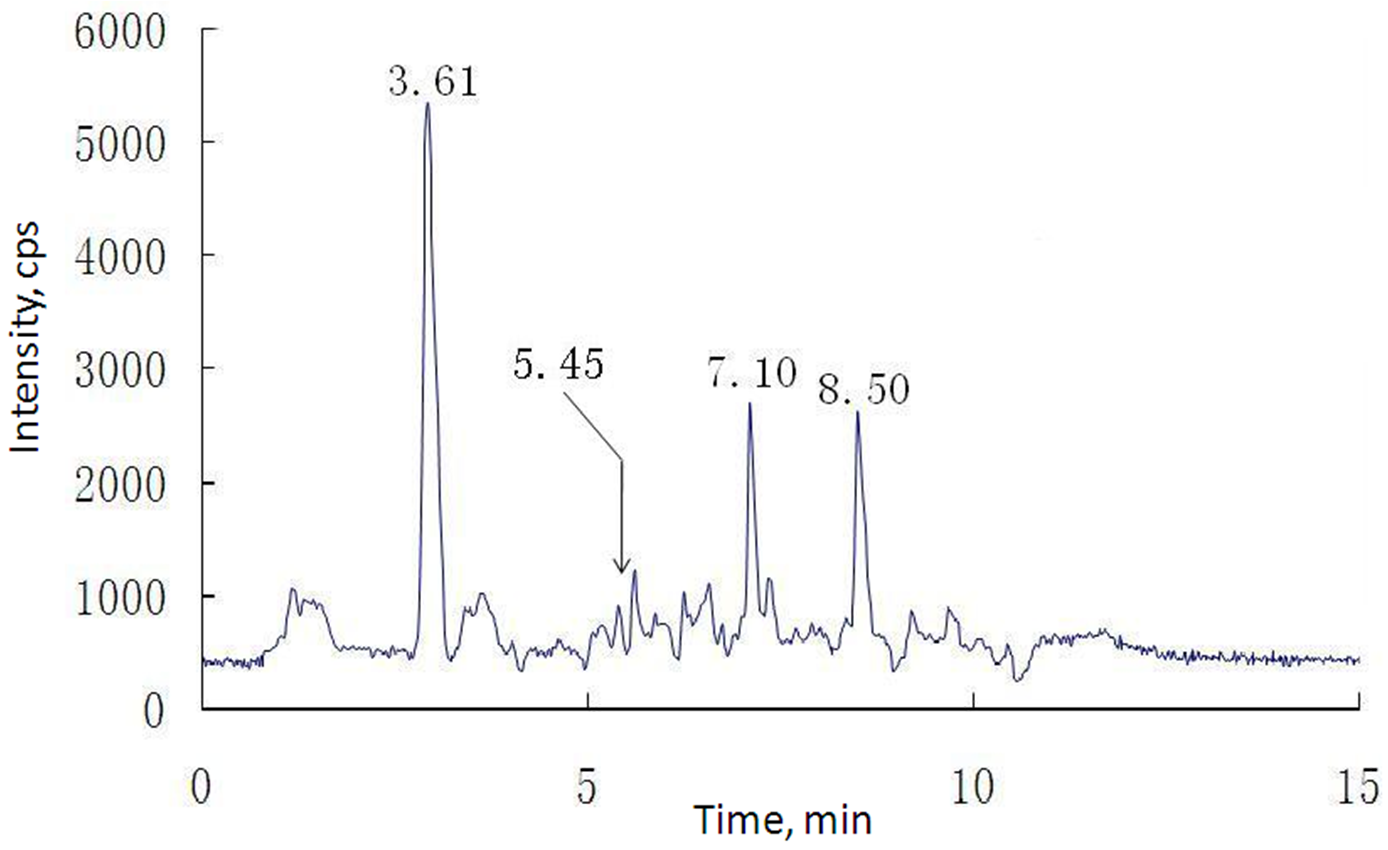

Supplement: Figure S6 — Chromatogram of testosterone metabolite in the medium before cell culture. It can be seen that no metabolite was found before incubation. (TIF) [file pone.0052255.s006.tif]

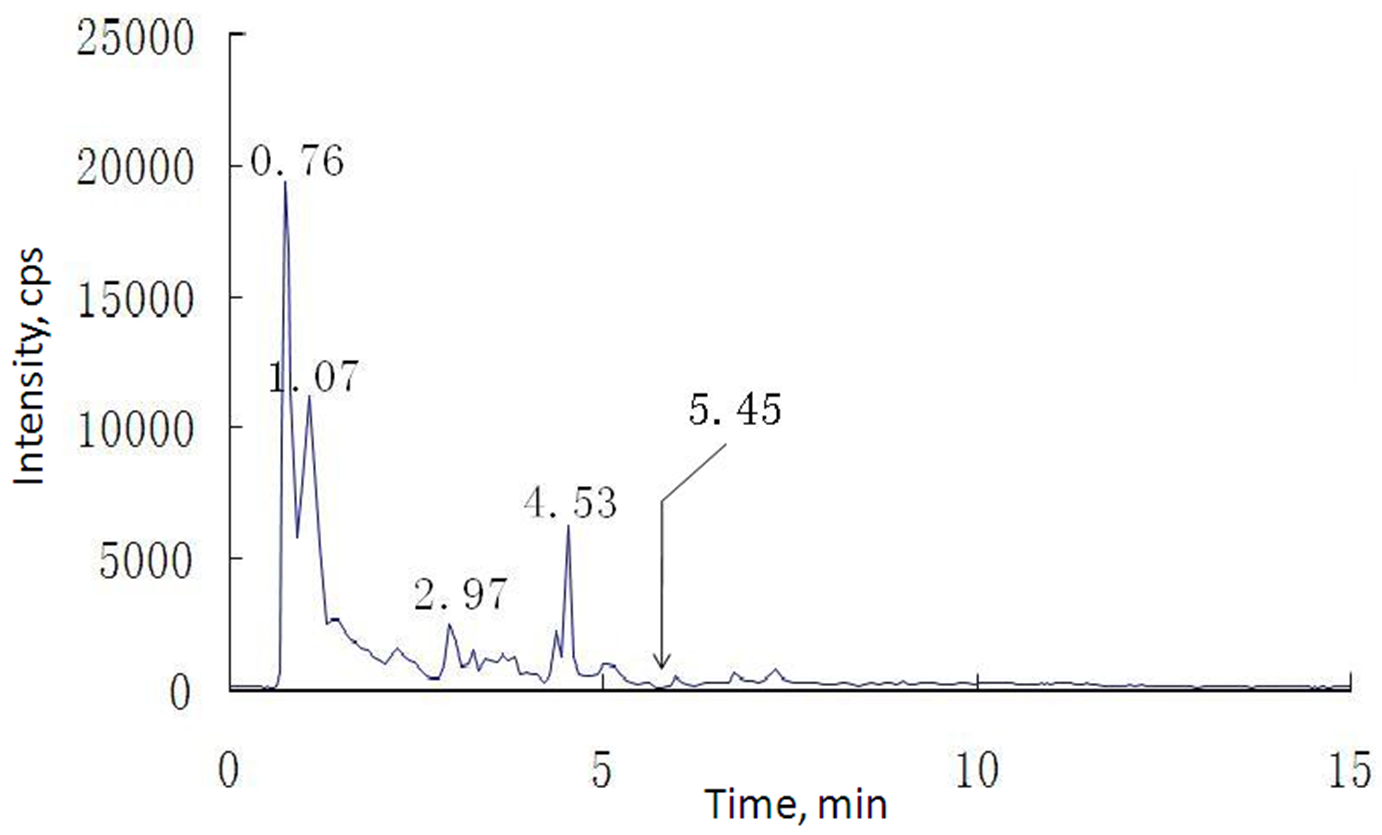

Supplement: Figure S7 — Chromatogram of testosterone metabolite in the medium without testosterone after cell culture. It can be seen that no metabolite was found in the blank medium. (TIF) [file pone.0052255.s007.tif]

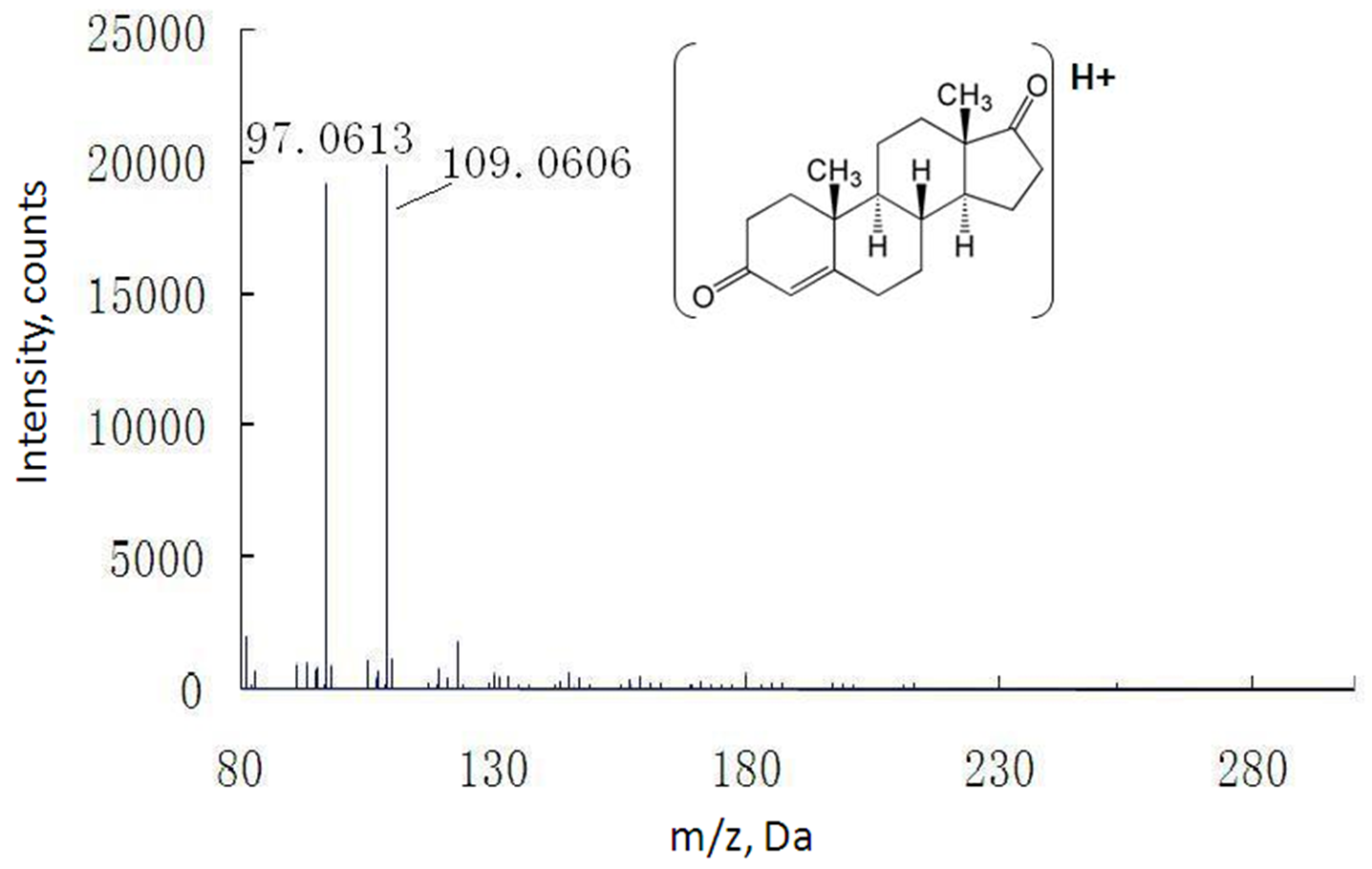

Supplement: Figure S8 — Product spectra of identified testosterone metabolite in the medium after cell culture. (TIF) [file pone.0052255.s008.tif]

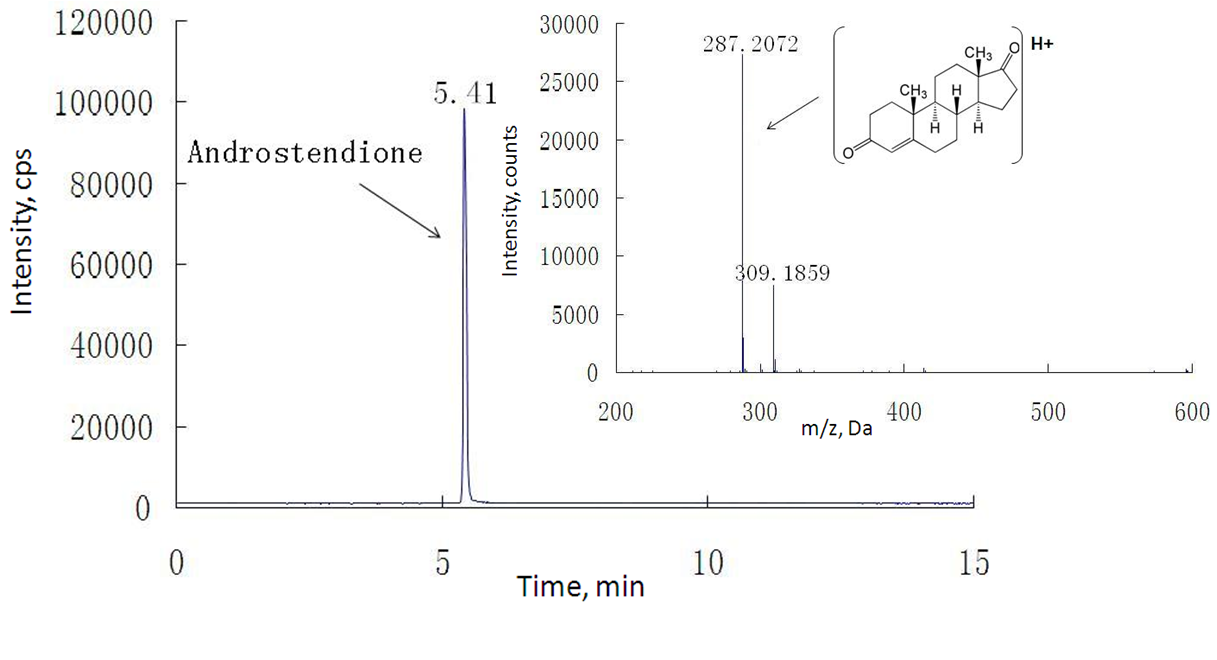

Supplement: Figure S9 — Chromatogram and mass spectra of androstendione standard in pure solvent. It can be seen that the chromatogram and mass spectra of testosterone metabolite match that of the androstendione standard. (TIF) [file pone.0052255.s009.tif]

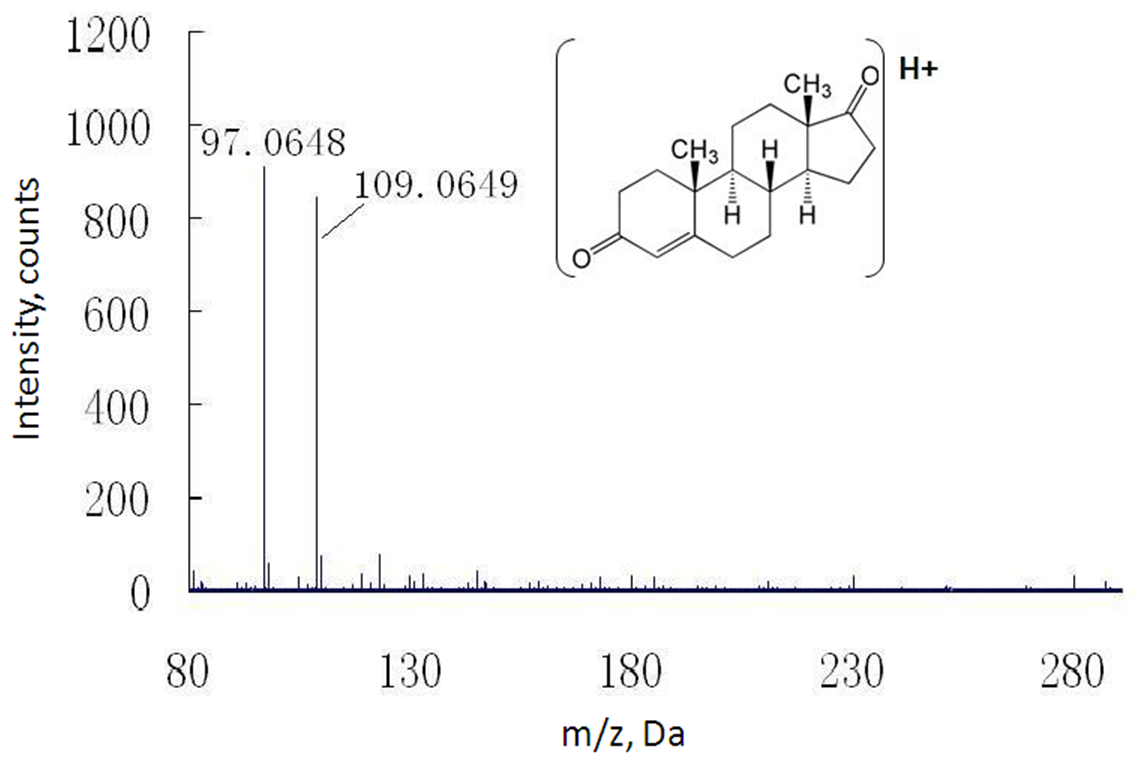

Supplement: Figure S10 — Product spectra of androstendione standard in pure solvent. The product ion pattern of testosterone metabolite matches that of the androstendione standard. (TIF) [file pone.0052255.s010.tif]

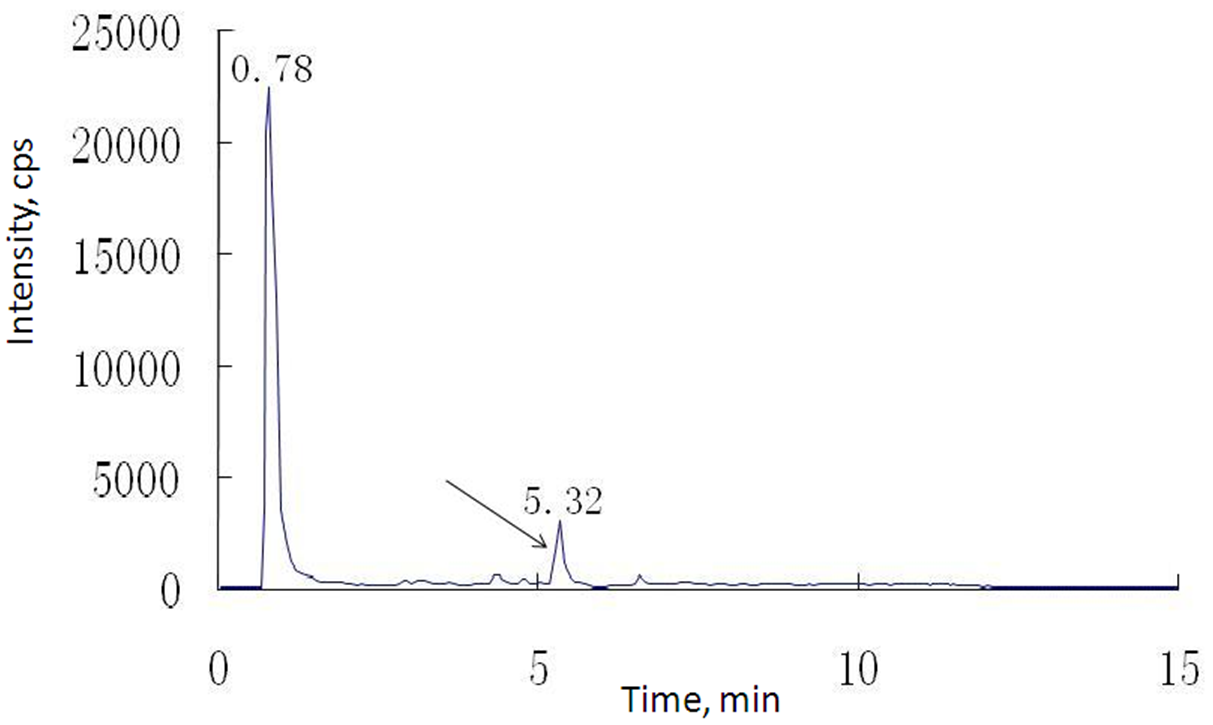

Supplement: Figure S11 — Chromatogram of β-estradiol in the medium after cell culture. It can be seen the β-estradiol level reduced more than 99% (comparing from peak height) after 24 hr cell culture. (TIF) [file pone.0052255.s011.tif]

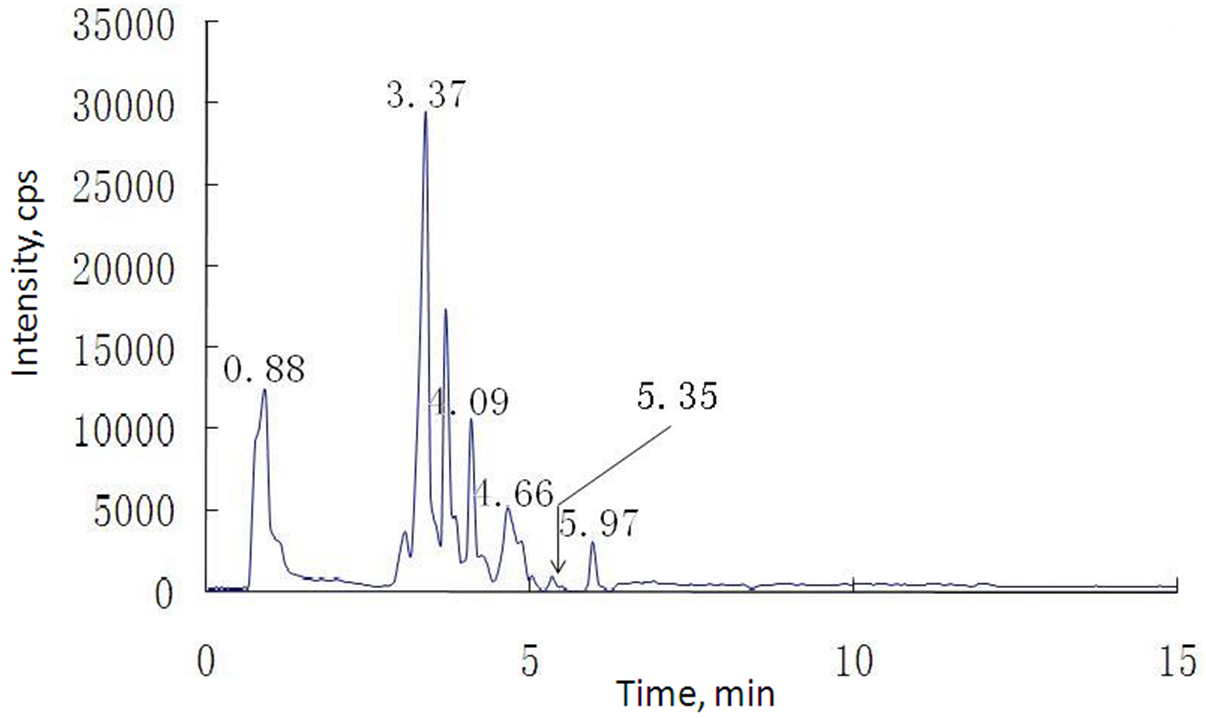

Supplement: Figure S12 — Chromatogram of β-estradiol metabolite in the medium before cell culture. It can be seen that no metabolite was found before incubation. (TIF) [file pone.0052255.s012.tif]

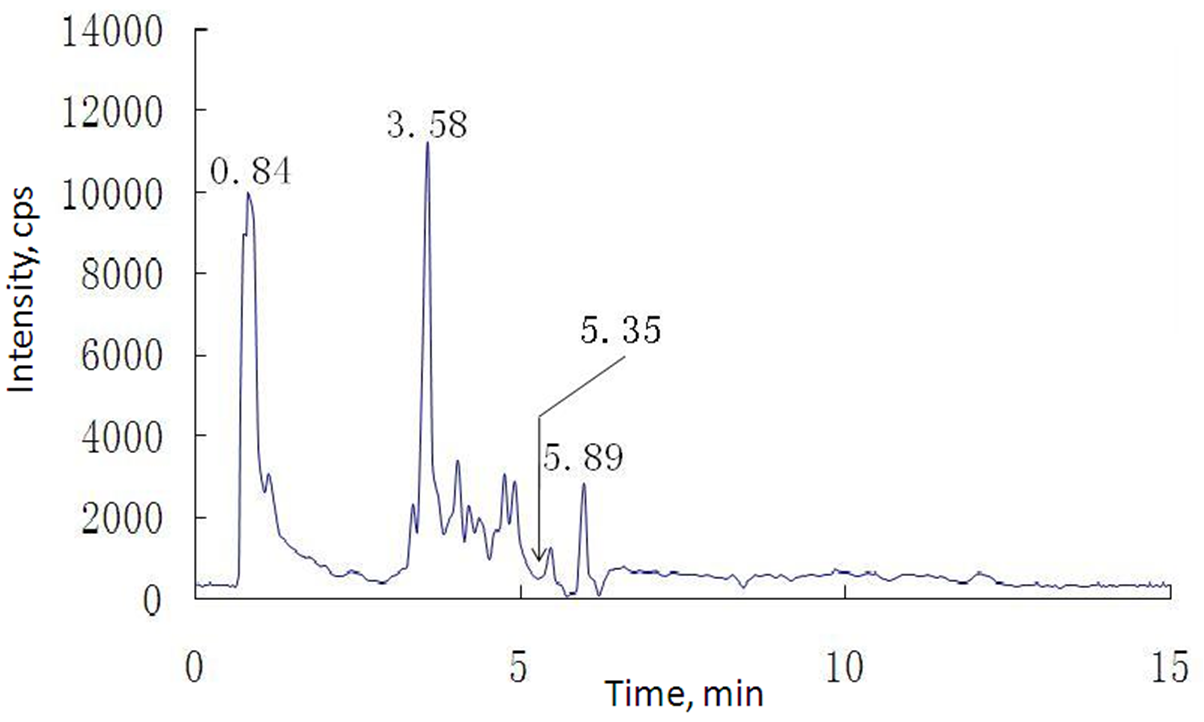

Supplement: Figure S13 — Chromatogram of β-estradiol metabolite in the medium without β-estradiol after cell culture. It can be seen that no metabolite was found in the blank medium. (TIF) [file pone.0052255.s013.tif]

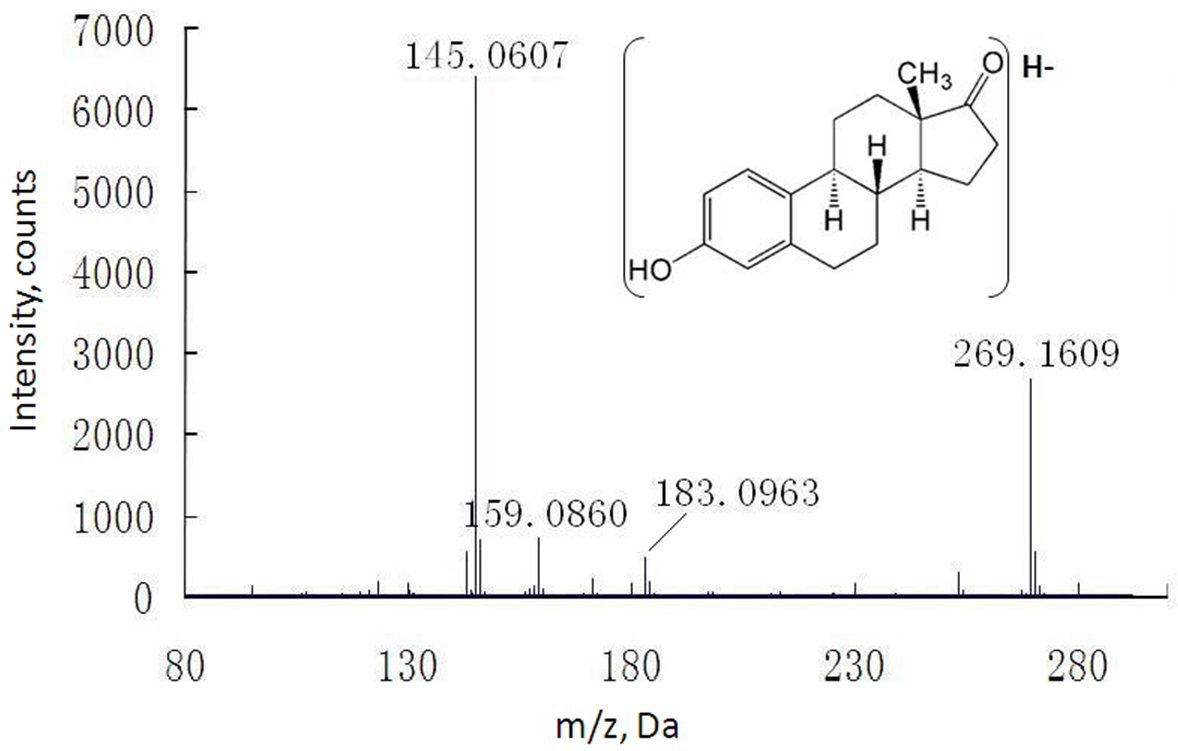

Supplement: Figure S14 — Product spectra of identified β-estradiol metabolite in the medium after cell culture. (TIF) [file pone.0052255.s014.tif]

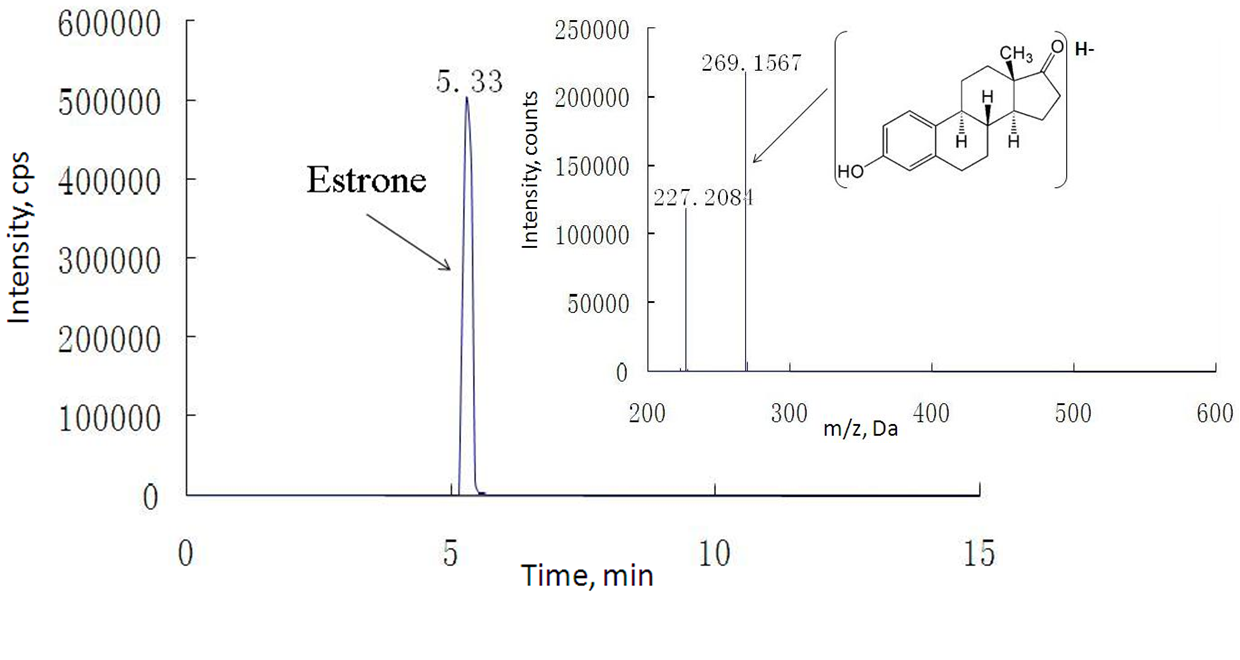

Supplement: Figure S15 — Chromatogram and mass spectra of estrone standard in pure solvent. It can be seen that the chromatogram and mass spectra of β-estradiol metabolite match that of the estrone standard. (TIF) [file pone.0052255.s015.tif]

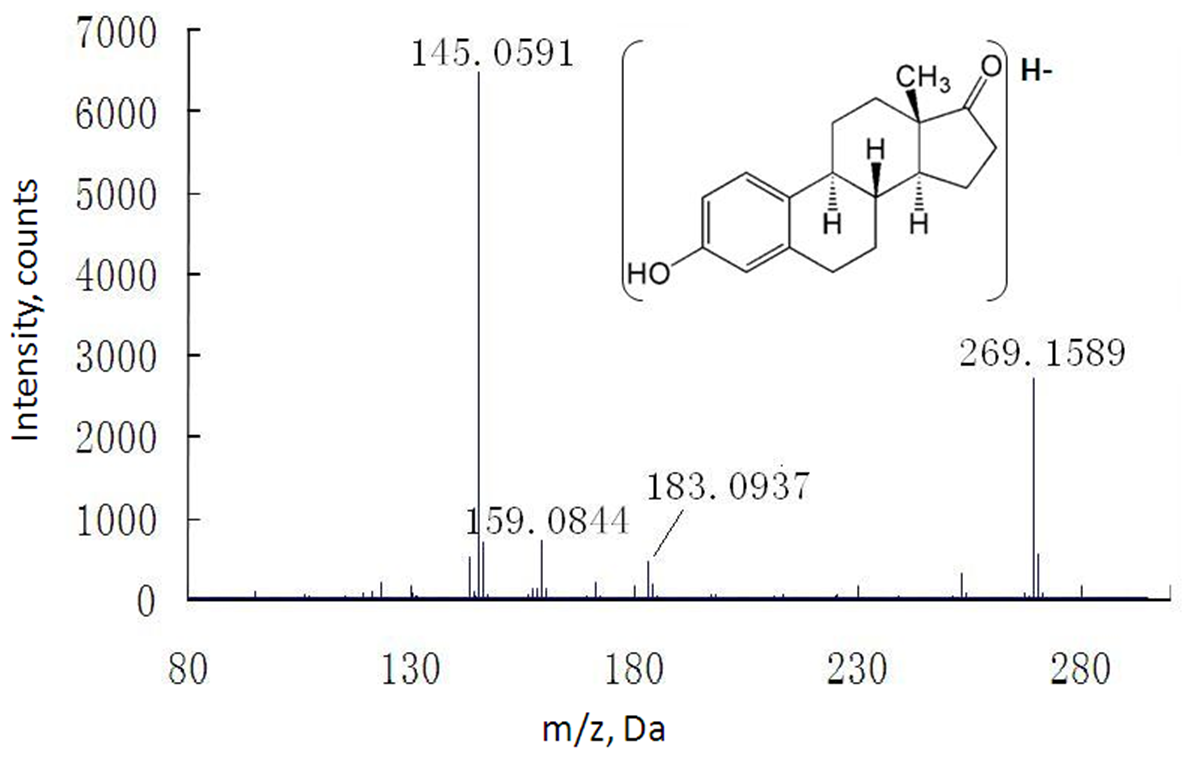

Supplement: Figure S16 — Product spectra of estrone standard in pure solvent. The product ion pattern of β-estradiol metabolite matches that of the estrone standard. (TIF) [file pone.0052255.s016.tif]
