# Supplementary material for: Investigation of Testosterone, Androstenone, and Estradiol Metabolism in HepG2 Cells and Primary Culture Pig Hepatocytes and Their Effects on 17βHSD7 Gene Expression
Source: PLoS One. 2012 Dec 26;7(12):e52255. doi: 10.1371/journal.pone.0052255 (PMC3530596; doi:10.1371/journal.pone.0052255)
Supplement: Table S1 — QSTAR® Elite mass spectrometer parameters (DOC) [file pone.0052255.s017.doc]

Table S1. QSTAR® Elite mass spectrometer parameters

| Index | ESI + | ESI - |
| --- | --- | --- |
| GS1 (psi) | 60 | 60 |
| GS2 (psi) | 50 | 50 |
| Curtain Gas (psi) | 20 | 20 |
| IonSpray Voltage (V) | 5500 | -4200 |
| Temperature (℃) | 500 | 500 |
| Declustering Potential (V) | 60 | -60 |
| Focusing Potential (V) | 265 | -265 |
| Declustering Potential 2 (V) | 10 | -10 |
| Ion Release Delay | 6.0 | 6.0 |
| Ion Release Width | 5.0 | 5.0 |
