# Supplementary material for: Investigation of Testosterone, Androstenone, and Estradiol Metabolism in HepG2 Cells and Primary Culture Pig Hepatocytes and Their Effects on 17βHSD7 Gene Expression
Source: PLoS One. 2012 Dec 26;7(12):e52255. doi: 10.1371/journal.pone.0052255 (PMC3530596; doi:10.1371/journal.pone.0052255)
Supplement: Table S2 — API 5000 mass spectrometer parameters (DOC) [file pone.0052255.s018.doc]

Table S2. API 5000 mass spectrometer parameters

| Index | ESI + | ESI - |
| --- | --- | --- |
| GS1 (psi) | 70 | 65 |
| GS2 (psi) | 50 | 50 |
| Curtain Gas (psi) | 20 | 15 |
| Collision Gas | 5 | 5 |
| IonSpray Voltage (V) | 5500 | -4500 |
| Temperature (℃) | 500 | 500 |
| Entrance Potential | 10 | -10 |
